# Marijuana use and its association with unhealthy weight control and muscle-enhancing behaviors among sexual minority men in the United States: a cross-sectional analysis

**DOI:** 10.1186/s42238-026-00398-9

**Published:** 2026-02-06

**Authors:** Chiamaka Ibeh, Yunan Zhao, Alvin Tran

**Affiliations:** 1 University of New Haven, 300 Boston Post Rd, West Haven, CT 06516 USA; 2 New York University, 227 E 30Th St, New York, NY 10016 USA

**Keywords:** Marijuana use, Unhealthy weight control behaviors, Muscle-enhancing behaviors, Sexual minority men, Body image, Disordered eating

## Abstract

**Introduction:**

Following marijuana legalization in several U.S. states, motivations for its use have expanded, especially among marginalized populations. While prior research links marijuana use with disordered eating, little is known about this relationship within sexual minority groups, who are already at elevated risk for body dissatisfaction and unhealthy weight control behaviors. This study examines associations between marijuana use and unhealthy weight control and muscle-enhancing behaviors among sexual minority men in the U.S.

**Methods:**

We conducted a secondary analysis using data from the Men’s Body Project, a cross-sectional study on body image and health behaviors. The sample included sexual minority men across diverse backgrounds. Logistic regression was used to assess associations between marijuana use and seven behaviors considered risk factors linked to body image concerns: fasting, vomiting, laxative use, diet pill use, muscle-building supplement use, protein powder use, and anabolic steroid use.

**Results:**

Marijuana users had significantly greater odds of engaging in all measured behaviors. Adjusted odds ratios (AORs) ranged from 1.88 (fasting) to 3.12 (diet pill use), all with *p*-values < 0.001. Other notable associations included vomiting (AOR = 2.61), laxative use (AOR = 2.23), protein powder use (AOR = 2.60), and anabolic steroid use (AOR = 2.62).

**Conclusion:**

Our results suggest a significant association between marijuana use and elevated odds of engaging in unhealthy weight control and muscle-enhancing behaviors among sexual minority men. Findings highlight the need for tailored public health interventions addressing both substance use and body image within LGBTQ + communities.

## Introduction

Unhealthy weight control behaviors (UWCBs) involve weight control strategies used to reduce or maintain body weight that may be harmful to overall health, such as fasting, diet pill or laxative use, and self-induced vomiting (Weng 2022; Zhao et al. 2024). Empirical studies indicate that sexual minority men (SMM; e.g., gay, bisexual, and men who have sex with men) experience a relatively high prevalence of victimization and discrimination and, in turn, are at increased risk of engaging in unhealthy weight control behaviors and disordered eating (Pistella 2019; Sutin 2020) and marijuana use (Wheldon 2023). Beyond exposure to discrimination, intraminority stressors, including body image norms and appearance-related pressures within some gay male communities, along with processes described by objectification theory, may help explain why sexual minority men demonstrate distinct patterns of body image concerns compared to heterosexual men (Soulliard et al. 2025). Prior research suggests that, whereas heterosexual men are more often encouraged to prioritize muscularity, sexual minority men may experience simultaneous pressures toward both muscularity and thinness, a combination that has been associated with greater vulnerability to disordered eating and unhealthy weight control behaviors (Tran et al. 2023).

Current evidence suggests a generally positive association between marijuana use and UWCBs (Weng 2022; Korn et al. 2018; Vidot et al. 2016). Prior studies have reported higher engagement in unhealthy weight loss practices among marijuana users compared to non-users, although the strength and statistical significance of these associations have varied across samples, particularly among adolescent populations (Korn et al. 2018; Vidot et al. 2016). Notably, this body of research has not examined these associations among sexual minority populations or older adults.

Research suggests that individuals who engage in unhealthy weight control behaviors are at increased risk for other potentially health-compromising behaviors, including substance use (Simone 1982). While marijuana remains federally classified as an illicit substance in the United States, many states permit recreational use among adults, and marijuana use nonetheless remains prevalent among adolescents and young adults (Jin 2017; Penner et al. 2013; Phillips 2018). Evidence indicates that marijuana-related policies, including legalization, may be associated with reduced perceptions of risk among adolescents and young adults and, in turn, higher levels of marijuana use at the population level, as reflected in increased cannabinoid detection in biological samples assessed through immunoassay-based surveillance studies (Tolan 2023; Park et al. 2024; Ryerson et al. 2024; Webert 2024). Long-term marijuana use is linked to broad decreases in brain activity (Webert 2024), verbal learning and memory disruption (Ajmera et al. 2021), and behavioral inhibition and decision-making inhibition (Testai et al. 2022). Collectively, these findings suggest that marijuana use may be associated with changes in cognitive and behavioral functioning, underscoring the importance of examining its co-occurrence with other health-related behaviors.

Individuals have various reasons and motivations for using marijuana. For example, some use marijuana to amplify pleasurable sensations (Berg 2018), including during sexual intercourse (Parent 2021), and marijuana use has been examined in relation to condomless anal sex among sexual minority youth (Cain 2021). Marijuana use has also been associated with mental health conditions such as depression (Dierker 2018), post-traumatic stress disorder (Jordan 2020), anxiety, and chronic pain (Sajdeya 2024). However, evidence regarding the analgesic effects of marijuana is mixed, with some studies and reviews indicating limited high-quality support for pain relief and the presence of adverse effects (Dahan 2024; Bicket et al. 2024).

Marijuana is known to stimulate appetite through activation of cannabinoid receptors, which has been associated with eating-related behaviors and appetite regulation (Lent et al. 2022). Prior research has reported associations between higher body mass index and marijuana and other substance use, although the directionality of these relationships remains unclear (Lanza 2022). Among young adults in the U.S. who perceive themselves as overweight, substance use and depressive symptoms have been associated with suicidal thoughts (Seo 2013). Peer perceptions of marijuana use (Barnett 2022) and parental marijuana use (Augustyn 2020; Madras 2019) are both positively correlated with marijuana use among emerging adults, highlighting the role of social and familial contexts in marijuana use behaviors. Lower levels of family support have been linked to greater involvement in peer-related risk behaviors. Reduced parental supervision (Prins 2021) and interpersonal violence, such as family physical violence and bullying, have been significantly associated with the adoption of unhealthy weight control practices (Okada et al. 2019).

Studies have suggested that adolescents who experience weight discrimination tend to engage in more substance use (Klinck et al. 2020) and unhealthy weight control behaviors (Gordon 2023), especially bisexual youth and those who are racially discriminated (Caves 2025). Based on our review of the existing literature, we believe our research is among the first studies to specifically examine the association between marijuana use and UWCBs in sexual minority male populations. In light of this existing gap in the scientific literature, our study aims to assess the association between marijuana use and unhealthy weight control and muscle-enhancing behaviors in a sample of diverse sexual minority men in the U.S.

## Methods

### Participants and recruitment

We conducted a secondary analysis using data from the Men’s Body Project (MBP), a cross-sectional online survey conducted in spring 2020 to examine health behaviors among gay and bisexual men in the United States. The parent study was approved by the University of New Haven Institutional Review Board (Protocol #2020–015), and data used for the present analysis were deidentified to the research team. All participants provided informed consent prior to survey completion.

Participants were recruited through Qualtrics Survey Panels, an online market research panel service. Panel members who met eligibility criteria were invited by Qualtrics to participate in the survey. Eligible participants included cisgender gay and bisexual men aged 18–50 years, residing in the United States, and able to read and understand English. Participants who completed the survey received incentives administered by Qualtrics (e.g., cash, airline miles, or vouchers) in accordance with its panel policies.

A total of 549 participants completed the survey. For the present secondary analysis, all participants with non-missing data on marijuana use and the outcomes of interest (unhealthy weight control behaviors and muscle-enhancing behaviors) were included, resulting in a final analytic sample of 549 participants.

### Measures

#### Marijuana use

The participants reported their marijuana use, indicating the number of times in a month they used marijuana over the past 12 months. Responses were dichotomized for analysis as ‘Never’ vs. all other frequencies.

### Unhealthy weight control behaviors and muscle-enhancing behaviors

Unhealthy weight control behaviors (UWCBs) were assessed using the following question: *“In the past year, how often did you do any of the following to lose weight or keep from gaining weight (fasting, self-induced vomiting, laxative use, or diet pill use)?”* The term *unhealthy weight control behaviors* is used to refer to these specific behaviors, which have been associated with adverse physical and psychological outcomes in prior research.

Muscle-enhancing behaviors were assessed separately and refer to behaviors aimed at increasing body mass or altering body shape or size rather than reducing body weight. These behaviors were evaluated using the question: *“In the past year, how often did you do any of the following (muscle-building supplements, protein powders, anabolic steroids) to maintain or change your body weight or shape?” *The wording and structure of the survey items used to assess UWCBs were adapted from the Youth Risk Behavior Surveillance System (YRBSS) survey (CDC. 2025). For analytic purposes, responses to all behavior items were dichotomized as 0 (never) and 1 (any other frequency).

### Sociodemographic and health-related covariates

Participants reported sociodemographic and health-related characteristics, including age, race, ethnicity, sexual orientation, relationship status, employment status, and cigarette smoking in the past 12 months. Body mass index (BMI) was calculated using self-reported height and weight. Age was categorized as 18–24 years, 25–34 years, and 35–50 years. Ethnicity was categorized as Hispanic or non-Hispanic, and race was categorized as White, Black, Asian/Pacific Islander, or American Indian/Other. Sexual orientation was categorized as gay or bisexual. Relationship status was categorized as single/dating, living with a partner, married/engaged, or divorced/widowed/separated/other. BMI was grouped into the following categories: < 18.5 kg/m^2^, 18.5–24.9 kg/m^2^, 25.0–29.9 kg/m^2^, and ≥ 30.0 kg/m^2^. Employment status was categorized as full-time, part-time, student, unemployed, or other. Cigarette smoking in the past 12 months was coded as yes (1) or no (0).

### Statistical analysis

Descriptive statistics were employed to examine the distribution of the study variables, providing an overview of their means, standard deviations, and frequency distributions, depending on whether the variables were categorical or continuous. Chi-square (χ2) tests and Student’s t-tests were performed for categorical variables and continuous variables, respectively, to examine differences between marijuana users and non-users. These tests were performed to obtain insights and compare the relevant differences in UWCBs between marijuana users and non-users.

We also conducted logistic regression models to assess the associations between UWCBs and muscle-enhancing behaviors among marijuana users and non-users. The adjusted model accounted for additional variables, including age, race, ethnicity, BMI, sexual orientation, relationship status, and employment status. However, we chose not to adjust for cigarette smoking status in the model because of the strong correlation between cigarette smoking and marijuana use (*p* < 0.001). Given that both behaviors are influenced by shared underlying factors rather than a causal relationship as opposed to cigarette smoking directly leading to marijuana use, adjusting for cigarette smoking could introduce bias or obscure the true relationship between marijuana use and the outcomes of interest.

## Results

The demographic characteristics of the study sample are presented in Table 1, which includes 549 male participants categorized into marijuana users (*n* = 237) and non-users (*n* = 312). Notably, 54.6% of the participants were in the 35–50 years age range. The racial makeup revealed that White participants accounted for the majority (71.4%), followed by Black (13.8%), Asian/Pacific Islander (8.0%), and American Indian/Other (6.7%) participants. With respect to sexual orientation, the sample consisted of slightly more gay participants (52.1%) than bisexual (47.9%). Approximately 40% of the participants had a BMI between 18.5 and 24.9 kg/m^2^, 34.8% had BMI between 25.0 and 29.9 kg/m^2^, and approximately 21.3% were ≥ 30.0 kg/m^2^. Additionally, we noted a strong association in cigarette smoking status between marijuana users and non-users (*p* value < 0.001).

**Table 1:** Sociodemographic characteristics of marijuana users and non-users (*N*=549)

	**Marijuana Users (*N* =237)**	**Non-users (*N* =312)**	**Overall (*N* =549)**	***p*-value**
Age (years)				< 0.001*
18–24	51 (21.5%)	56 (17.9%)	107 (19.5%)	
25–34	83 (35.0%)	59 (18.9%)	142 (25.9%)	
35–50	103 (43.5%)	197 (63.1%)	300 (54.6%)	
Ethnicity				0.120
Hispanic	52 (21.9%)	51 (16.3%)	103 (18.8%)	
Non-Hispanic	185 (78.1%)	261 (83.7%)	446 (81.2%)	
Race				0.015*
White	162 (68.4%)	230 (73.7%)	392 (71.4%)	
Black	43 (18.1%)	33 (10.6%)	76 (13.8%)	
Asian/Pacific Islander	13 (5.5%)	31 (9.9%)	44 (8.0%)	
American Indian/Other	19 (8.0%)	18 (5.8%)	37 (6.7%)	
BMI (kg/m2)				0.472
<18.5	11 (4.6%)	11 (3.5%)	22 (4.0%)	
18.5 − 24.9	102 (43.0%)	117 (37.5%)	219 (39.9%)	
25.0 − 29.9	77 (32.5%)	114 (36.5%)	191 (34.8%)	
≥ 30.0	47 (19.8%)	70 (22.4%)	117 (21.3%)	
Sexual Orientation				0.086
Gay	113 (47.7%)	173 (55.4%)	286 (52.1%)	
Bisexual	124 (52.3%)	139 (44.6%)	263 (47.9%)	
Relationship Status				0.568
Single/Dating	125 (52.7%)	182 (58.3%)	307 (55.9%)	
Living with a partner	35 (14.8%)	45 (14.4%)	80 (14.6%)	
Married/engaged	65 (27.4%)	72 (23.1%)	137 (25.0%)	
Divorced/Widowed/Separated/Other	12 (5.1%)	13 (4.2%)	25 (4.6%)	
Employment Status				0.511
Full-time	149 (62.9%)	198 (63.5%)	347 (63.2%)	
Part-time	23 (9.7%)	26 (8.3%)	49 (8.9%)	
Student	21 (8.9%)	30 (9.6%)	51 (9.3%)	
Unemployed	31 (13.1%)	49 (15.7%)	80 (14.6%)	
Other	13 (5.5%)	9 (2.9%)	22 (4.0%)	
Smoke (past 12 months)				<0.001*
Yes	145 (61.2%)	80 (25.6%)	225 (41.0%)	
No	92 (38.8%)	232 (74.4%)	324 (59.0%)	

*Indicates a *p*-value <0.05. Chi-Square (χ2) tests and student’s t-test were performed for categorical variables and continuous variables, respectively, to examine differences between marijuana users and non-users

The data obtained from Table 2 presents the differences between UWCBs, muscle-building supplement use, protein powder use, and anabolic steroid use among marijuana users and non-users. Analysis via chi-square tests revealed a significant association between UWCBs, muscle-building supplements, protein powders and anabolic steroid use in marijuana users and non-users (*p* values < 0.001). Across all the behaviors, marijuana users reported significantly greater engagement in UWCBs than non-users did (*p* < 0.001 for all the comparisons). Specifically, fasting was reported by 51.5% of marijuana users versus 33.0% of non-users, vomiting by 31.2% versus 13.1%, and laxative use by 32.1% versus 14.7%. Similarly, diet pill use was more prevalent among marijuana users (35.4%) than non-users (13.5%), as was the use of muscle-building supplements (40.1% vs. 19.6%), and protein powder (53.6% vs. 27.9%). Protein powder use and fasting were the most frequently reported among marijuana users, with rates of 53.6% and 51.5%, respectively, compared with 27.9% and 33%, respectively, among non-users. The least prevalent behavior is anabolic steroids use, with rates of 28.7% and 11.2% among marijuana users and non-users, respectively.

**Table 2:** Unhealthy weight control behaviors and muscle-enhancing behaviors among marijuana users and non-users (*N* = 549)

	**Marijuana Users (** ***N*** ** =237)**	**Non-users (** ***N*** ** =312)**	**Overall (** ***N*** ** =549)**	***p*** **-value**
Fasting	122 (51.5%)	103 (33.0%)	225 (41.0%)	<0.001*
Vomiting	74 (31.2%)	41 (13.1%)	115 (20.9%)	<0.001*
Laxative use	76 (32.1%)	46 (14.7%)	122 (22.2%)	<0.001*
Diet pill use	84 (35.4%)	42 (13.5%)	126 (23.0%)	<0.001*
Use of Muscle-building supplements^a^	95 (40.1%)	61 (19.6%)	156 (28.4%)	<0.001*
Protein powder use	127 (53.6%)	87 (27.9%)	214 (39.0%)	<0.001*
Anabolic steroid use	68 (28.7%)	35 (11.2%)	103 (18.8%)	<0.001*

*Indicates a *p*-value <0.05. Chi-Square (χ2) tests and student’s t-test were performed for categorical variables and continuous variables, respectively, to examine differences between marijuana users and non-users. ^a^ Includes products such as creatine, amino acids, DHEA, hydroxyl methyl-butyrate [HMB], or growth hormone

Table 3 highlights that marijuana users were significantly more likely than non-users to engage in a wide range of unhealthy weight control and muscle-enhancing behaviors. In the unadjusted analyses, marijuana use was associated with more than twice the odds of engaging in behaviors such as fasting, vomiting, laxative use, diet pill use, muscle-building supplement use, protein powder use, and anabolic steroid use, with odds ratios ranging from 2.15 to 3.53, all statistically significant at *p* < 0.001. These associations remained significant even after adjusting for potential confounding variables. Adjusted odds ratios ranged from 1.88 to 3.12, indicating a statistically significant association between marijuana use and higher odds of each behavior assessed. Notably, the strongest associations were observed for diet pill use (AOR = 3.12), anabolic steroid use (AOR = 2.62), and vomiting (AOR = 2.61), suggesting that marijuana use may be particularly linked to more extreme weight and body shape control strategies. Overall, these findings indicate a significant association between marijuana use and increased engagement in UWCBs and muscle-enhancing behaviors.

**Table 3:** Odds of UWCBs and muscle-enhancing behaviors among marijuana users and non-users (*N*=549)

	Unadjusted		Adjusted^b^	
	Odds Ratio [95% CI]	*p* value	Odds Ratio [95% CI]	*p* value
Fasting	2.15 [1.52, 3.05]	<0.001*	1.88 [1.29, 2.75]	<0.001*
Vomiting	3.00 [1.97, 4.64]	<0.001*	2.61 [1.59, 4.32]	<0.001*
Laxative use	2.73 [1.81, 4.16]	<0.001*	2.23 [1.40, 3.58]	<0.001*
Diet pill use	3.53 [2.33, 5.41]	<0.001*	3.12 [1.95, 5.05]	<0.001*
Use of Muscle-building supplements ^a^	2.75 [1.88, 4.05]	<0.001*	2.35 [1.54, 3.63]	<0.001*
Protein powder use	2.99 [2.10, 4.27]	<0.001*	2.60 [1.75, 3.88]	<0.001*
Anabolic steroid use	3.18 [2.04, 5.04]	<0.001*	2.62 [1.57, 4.43]	<0.001*

*Indicates a p-value < 0.05; Reference group are non-users of marijuana

^a^Includes products such as creatine, amino acids, DHEA, hydroxyl methyl-butyrate [HMB], or growth hormone

^b^Adjusted for demographic characteristics include age, race, ethnicity, BMI, sexual orientation, relationship status, and employment status

## Discussion

Marijuana users exhibited consistently greater odds of engagement across all UWCBs and muscle-enhancing behaviors assessed, including fasting, vomiting, laxative use, diet pill use, body-building supplements, protein powder, and anabolic steroid use, in both the adjusted and unadjusted analyses. The results of our study align with those of other researchers who have examined the relationship between marijuana use and UWCBs (Korn et al. 2018; Vidot et al. 2016). Vidot and colleagues (Vidot et al. 2016) reported that Florida high school students who used substances, including marijuana, alcohol, and cigarettes, were significantly more likely to engage in unhealthy weight loss practices than those who did not use marijuana. Meanwhile, Korn and colleagues (Korn et al. 2018) suggested that frequent marijuana use (20 + times in the past year) was significantly associated with an increased likelihood of engaging in UWCBs. Their findings indicated that frequent marijuana users had 55% greater odds of engaging in UWCBs than non-users, even after controlling for other factors. However, both studies exclusively examined teenagers, particularly girls and emerging adults. To our knowledge, no recent research has focused specifically on older adults and SMM.

Anxiety related to weight and body image may be associated with engagement in UWCBs. Prior research indicates that weight perception and body dissatisfaction are strongly correlated with both healthy and unhealthy weight control practices, with anxiety and dissatisfaction more consistently linked to unhealthy approaches (Barinas 2022). The associated anxiety may drive individuals away from time-consuming healthy methods such as exercising and toward unhealthy methods for weight control perceived as easier. Body image concerns among minority populations have also been associated with higher prevalence of eating disorders (McLean and Paxton 2019), poor mental health, harmful body change behaviors (Rodgers 2023), and greater substance use (Rodgers 2021).

These patterns may reflect the cumulative effects of minority stress, including distal stressors (e.g., experiences of bias, bullying, and discrimination) and proximal stressors (e.g., internalized stigma and heightened vigilance), which are commonly experienced by sexual minority men (Frost 2023). Such stressors may contribute to psychological distress and heightened body image concerns. Prior research has reported that gay and bisexual men are less likely to engage in certain forms of physical or strength-building activities compared to their heterosexual counterparts (Laska et al. 2015); however, this does not necessarily imply greater sedentary behavior or higher body mass index. Rather, differences in body ideals, appearance norms, and social contexts may shape engagement in unhealthy weight control and muscle-enhancing behaviors independent of overall physical activity levels. A sedentary lifestyle, characterized by low levels of physical activity, has been associated with increased substance use (West et al. 2020) and marijuana use (Jin 2017), as well as eating disorders, such as anorexia nervosa (Rogers 2023).

While marijuana is known to stimulate appetite through activation of cannabinoid receptors, its overall impact on body weight remains unclear. The relationship between marijuana use and body mass index is inconsistent across studies. Some research has found no significant association between marijuana use and obesity (Romano 2023; Scheffler et al. 2018). Similarly, a study of individuals with obesity initiating medical marijuana treatment for chronic pain and anxiety observed no notable weight changes during the first three months of use (Bicket et al. 2024). Other research suggests that marijuana use may be associated with lower BMI, both among psychologically stable individuals (Alshaarawy 2019) and among those with psychosis (Vázquez-Bourgon 2019). Chronic marijuana users, compared with non-users, have also been reported to exhibit lower BMI and more favorable metabolic profiles, including reduced glucose, insulin, cholesterol, and lipoprotein levels (Penner et al. 2013; Kramer et al. 2020; Le Strat and Le Foll 2011). In contrast, some studies have reported higher BMI among adolescents who use cannabis (Ross et al. 2016). Taken together, these findings indicate that further research is needed to clarify the relationship between marijuana use and body weight.

### Implications for future research

Because the present study was cross-sectional and did not assess causal relationships, future research employing prospective, longitudinal designs is needed to clarify the temporal ordering and directionality of associations between marijuana use and unhealthy weight control and muscle-enhancing behaviors. Longitudinal studies following marijuana users and non-users over time could help determine how these behaviors emerge, co-occur, and change across the life course. In addition to quantitative approaches, qualitative research methods, such as in-depth interviews or focus groups, may provide deeper insight into the lived experiences, social contexts, and motivations underlying marijuana use and body-related behaviors, particularly among sexual minority men.

Future studies should also explore how intersecting factors such as race, ethnicity, socioeconomic status, and gender identity influence these relationships. Examining these dynamics through an intersectional lens can help identify subgroup-specific risk factors and tailor interventions accordingly. Furthermore, given the variety of marijuana products on the market, research should investigate whether different modes of consumption (e.g., vaping, edibles, smoking) or potency levels are differentially associated with UWCBs and muscle-enhancing behaviors.

Mental health may also play a critical mediating or moderating role in these associations. Exploring the impact of conditions such as anxiety, depression, or trauma could provide a more comprehensive understanding of how marijuana use and disordered eating behaviors are connected. Additionally, studies should consider how broader environmental and policy-level factors, including the availability and legal status of marijuana, shape these behaviors within LGBTQ + communities.

### Limitations

This study has several limitations that should be considered when interpreting the findings. First, the analysis is based on secondary data from the Men’s Body Project, which limited our ability to influence the original study design, survey content, or data collection procedures. All measures were self-reported, introducing the potential for recall bias and social desirability bias, particularly for sensitive behaviors such as marijuana use and unhealthy weight control practices. Second, the cross-sectional nature of the study precludes any conclusions about causality. While we observed associations between marijuana use and unhealthy weight control and muscle-enhancing behaviors, we cannot determine the temporal sequence of these behaviors or infer cause-and-effect relationships. Because multiple statistical tests were conducted, there is an increased risk of type I error; findings should therefore be interpreted cautiously and viewed as exploratory. Third, although we adjusted for participants’ employment status, we did not have a comprehensive measure of socioeconomic status, which has been associated with marijuana use (Jeffers 2021) and may represent an unmeasured confounder in the observed associations with unhealthy weight control and muscle-enhancing behaviors. Fourth, marijuana use was dichotomized to distinguish any past-year use from no use, which limited our ability to assess frequency- or dose–response relationships and reduced comparability with studies that define frequent use using higher thresholds (e.g., ≥ 20 uses per year). Fifth, we did not adjust for cigarette smoking in the primary models due to its high correlation with marijuana use, which may introduce residual confounding. Finally, the generalizability of the findings is limited by the sample composition. The study focused exclusively on cisgender gay and bisexual men aged 18–50 years residing in the U.S. and recruited via online survey panels. However, despite these limitations, this study provides important and timely insights into a largely understudied area. To our knowledge, it is among the first to specifically examine the association between marijuana use and unhealthy weight control and muscle-enhancing behaviors in a national sample of sexual minority men.

## Conclusion

Our findings indicate a significant association between marijuana use and engagement in unhealthy weight control and muscle-enhancing behaviors among sexual minority men in the U.S. These findings highlight the importance of developing integrated intervention strategies and health education programs that consider the co-occurrence of marijuana use and weight control-related behaviors, particularly within the sociocultural context of sexual minority men. Lastly, through focusing on a population disproportionately affected by body image concerns and substance use, the study contributes to a growing body of literature highlighting the intersection of mental health, identity-based stress, and health behaviors. The findings underscore the need for targeted, culturally responsive public health interventions that address both substance use and disordered eating within LGBTQ + communities.

## Data Availability

The datasets used and/or analysed during the current study are available from the corresponding author on reasonable request.

## Abbreviations

UWCBs: Unhealthy weight control behaviors
SMM: Sexual minority men
MBP: Men’s body project
BMI: Body mass index
AOR: Adjusted odds ratio
CI: Confidence interval
U.S.: United States
